# What Happens to Patients' Leavings?

**Published:** 1919-07-05

**Authors:** Gladys M. E. Leigh


*To the Editor of* THE HOSPITAL.

### What Happens to Patients' Leavings?

*To the Editor of* THE HOSPITAL.

SIR,—It would be quite possible, with very little additional expense, in hospitals where sufficient ground space is available, for both poultry and pigs to be reared on the unavoidable waste of both cooked and uncooked food which is bound to occur in large institutions. Taking into consideration the high cost of eggs, bacon, and poultry, a distinct economy would be effected. In congested areas surely a scheme would be practicable for collecting from the various institutions their leavings, for conveyance to a central depôt where the fat could be extracted and converted into lubricants?

Yours faithfully,

GLADYS M. E. LEIGH.

[A former correspondent has already mentioned the pig. Another, for hospitals with no ground, has suggested that leavings can be sold to pig-farmers. Is there no alternative?—ED. THE HOSPITAL.]

